# Complete Genome Sequence of a Coastal Cyanobacterium, *Synechococcus* sp. Strain NIES-970

**DOI:** 10.1128/genomeA.00139-17

**Published:** 2017-04-06

**Authors:** Yohei Shimura, Yuu Hirose, Naomi Misawa, Sachiko Wakazuki, Takatomo Fujisawa, Yasukazu Nakamura, Yu Kanesaki, Haruyo Yamaguchi, Masanobu Kawachi

**Affiliations:** a Center for Environmental Biology and Ecosystem Studies, National Institute for Environmental Studies, Tsukuba, Ibaraki, Japan; b Department of Environmental and Life Sciences, Toyohashi University of Technology, Toyohashi, Aichi, Japan; c Electronics-Inspired Interdisciplinary Research Institute (EIIRIS), Toyohashi University of Technology, Toyohashi, Aichi, Japan; d Center for Information Biology, National Institute of Genetics, Research Organization of Information and Systems, Mishima, Shizuoka, Japan; e NODAI Genome Research Center, Tokyo University of Agriculture, Tokyo, Japan

## Abstract

Members of the cyanobacterial genus *Synechococcus* are abundant in marine environments. To better understand the genomic diversity of marine *Synechococcus* spp., we determined the complete genome sequence of a coastal cyanobacterium, *Synechococcus* sp. NIES-970. The genome had a size of 3.1 Mb, consisting of one chromosome and four plasmids.

## GENOME ANNOUNCEMENT

*Synechococcus* is a morphologically defined genus composed of unicellular, spherical- to rod-shaped cyanobacteria (1) that have a polyphyletic origin (2). Marine *Synechococcus* spp. are distributed over a range of environments, from coastal to pelagic, and *Synechococcus* spp., which belong to the same clade as *Prochlorococcus* spp., are known to be the most common and dominant species in pelagic oceans (3). However, the genetic and biological diversities of coastal *Synechococcus* spp. consisting of polyphyletic groups are still not understood. Here, we present the genome sequence of *Synechococcus* sp. NIES-970, a coastal cyanobacterium strain isolated from a tidal mud flat at Rikuhama Beach, Tokunoshima Island, Kagoshima, Japan in 1998, and maintained in the Microbial Culture Collection at the National Institute for Environmental Studies (http://mcc.nies.go.jp).

A paired-end library and a mate-pair library of 8-kb inserts were prepared using a Nextera XT DNA library preparation kit (Illumina, San Diego, CA, USA) and a Nextera mate-pair sample preparation kit (Illumina), respectively. These libraries were sequenced on a MiSeq sequencer (Illumina) with the MiSeq Reagent kit version 3 (600 cycles; Illumina). The output reads were filtered based on a 17-mer frequency using ShortReadManager (4) and then *de novo* assembled using Newbler version 2.9 (Roche Applied Science, Penzberg, Germany), which yielded 16 large contigs (>500 bp) and seven scaffolds. The average depth of sequence coverage was calculated to be 185-fold. The gap sequences were determined *in silico* using GenoFinisher and AceFileViewer (4), and the genome was found to consist of five circular structures, probably one chromosome and four plasmids. The total genome size was 3,123,859 bp, and the G+C content was 49.4%. Prediction of protein-coding genes, rRNA genes, and tRNA genes using Prokka (5) estimated 2,943, six, and 44 genes, respectively. Annotations of the predicted protein-coding genes were then conducted using original computer scripts that refer to the manually curated annotations in CyanoBase (6).

*Synechococcus* sp. NIES-970 was closely related to *Synechococcus* sp. NKBG15041c (7), with a 16S rDNA sequence of NIES-970 that was completely identical to that of NKBG15041c. We conducted reciprocal protein BLASTs between deduced protein sequences of *Synechococcus* sp. NIES-970 and NKBG15041c using BLAST+ version 2.3.0 (8), and the bidirectional BLAST best-hit pairs with query coverage ≥90% and percentage of identical matches ≥50 were considered as orthologous. The number of orthologs was 2,628, accounting for 89.3% of deduced proteins in *Synechococcus* sp. NIES-970. Furthermore, the results of the reciprocal protein BLASTs revealed that *Synechococcus* sp. NIES-970 has a putative copper-resistance, gene-regulating, two-component sensor histidine kinase CopS (locus_tag; NIES-970_28860) and a response regulator CopR (locus_tag; NIES-970_28880), which were located on a plasmid; however, NKBG15041c does not have these orthologs. The BLAST identity scores of the putative CopS and CopR to those of *Synechocystis* sp. PCC 6803 (9) were only 47% and 63%, respectively; however, putative copper-resistance protein-coding genes (locus_tag; NIES-970_28920 and NIES-970_28930), which were also not found on the NKBG15041c genome, were located near the *copS* and *copR* genes. Based on the genetic differences, NIES-970 may have higher copper-resistance ability than NKBG15041c.

### Accession number(s).

The genome sequence of *Synechococcus* sp. NIES-970 has been deposited in DDBJ/EMBL/GenBank under the accession numbers AP017959 to AP017963.
